# CSRR-SICW High Sensitivity High Temperature Sensor Based on Si_3_N_4_ Ceramics

**DOI:** 10.3390/mi12040459

**Published:** 2021-04-19

**Authors:** Shujing Su, Ting Ren, Lili Zhang, Fujia Xu

**Affiliations:** Science and Technology on Electronic Test & Measurement Laboratory, North University of China, No.3 Xueyuan Road, Taiyuan 030051, China; ritty_a@126.com (T.R.); lilizhang8769@sina.cn (L.Z.); x1186159946@163.com (F.X.)

**Keywords:** CSRR-SICW, Si_3_N_4_ ceramics, high temperature sensor, high sensitivity, wireless

## Abstract

A new type of wireless passive, high sensitivity, high temperature sensor was designed to meet the real-time temperature test in the harsh aero-engine environment. The sensor consists of a complementary split ring resonator and a substrate integrated circular waveguide (CSRR-SICW) structure and is based on high temperature resistant Si_3_N_4_ ceramic as the substrate material. Temperature is measured by real-time monitoring of the resonant frequency of the sensor. In addition, the ambient temperature affects the dielectric constant of the dielectric substrate, and the resonant frequency of the sensor is determined by the dielectric constant, so the function relationship between temperature and resonant frequency can be established. The experimental results show that the resonant frequency of the sensor decreases from 11.3392 GHz to 11.0648 GHz in the range of 50–1000 °C. The sensitivity is 123 kHz/°C and 417 kHz/°C at 50–450 °C and 450–1000 °C, respectively, and the average test sensitivity is 289 kHz/°C. Compared with previously reported high temperature sensors, the average test sensitivity is approximately doubled, and the test sensitivity at 450–1000 °C is approximately three times higher. Therefore, the proposed high sensitivity sensor has promising prospects for high temperature measurement.

## 1. Introduction

The aero-engine is the heart of an aircraft [1]. In the process of aero-engine development, temperature is an important parameter for performance analysis, design verification and improvement, and flow heat transfer analysis [2]. The aero-engine is characterized by high temperature, high pressure, high speed, complex internal flow, complex structure, small space, etc., so temperature measurement under such working conditions has always been a hot issue in aviation test and test technology, and also one of the difficulties in aero-engine test technology [3]. In view of the complex environment inside the aero-engine, researchers have tried temperature measurement technology based on various principles.

Thin film thermocouple, radiation temperature sensing, and temperature indicator paint are the main temperature measurement methods used in aero-engines at present [4,5,6,7,8]. The thin film thermocouple designed in literature [4] basically eliminates the influence of embedded thermocouple on the measured temperature field. However, the thin film thermocouple is not suitable for large-scale installation due to a lead line problem, especially for the temperature measurement of high-temperature rotating parts of engines (such as turbine blades). In the literature [5,6], the temperature measuring crystal has characteristics of small size and no lead line, but it can only test the highest temperature in the transformation process, and cannot be applied to real-time temperature monitoring. [7,8] designed the thermopaint temperature measuring method, which is a non-interference temperature measurement method. As a functional paint whose color changes with temperature, thermopaint has the advantages of not destroying the target structure, not affecting the target temperature field, and intuitionistic results. While the measurement characteristics of thermopaint offer convenience, they also bring corresponding limitations. The temperature value needs to be read by color comparison, so the subjective error is large, and the irreversible thermopaint cannot be used repeatedly, but can only measure the maximum temperature of the target, which is difficult to meet the requirements of real-time and accurate temperature measurement.

Wireless passive sensing technology has incomparable advantages in obtaining temperature parameters in harsh environments, which has attracted the attention of researchers. Wireless passive temperature measurement technology mainly includes four kinds: surface acoustic wave (SAW), sensitive capacitance and inductance (LC) resonant mutual inductance coupling technology, optical fiber technology, and microwave backscattering technology [9,10]. The SAW sensor offers the advantages of simple structure, small volume, and long transmission distance, but is limited by substrate material instability at high temperatures, making it is easy for the test signal to be interfered by the environment [11]. The LC resonant sensor adopts near-field coupling technology, which has great advantages for short distance signal transmission, but its operating frequency is low, so it cannot be attached to the metal surface [12,13]. In addition to the advantages of wide temperature measuring range, high sensitivity, and good electromagnetic insulation, the optical fiber temperature sensor is mainly characterized by a temperature measuring probe with an optical fiber. As a small probe needs to be placed under high temperature and high speed gas flow for contact measurement, a large number of experiments are needed for verification and assessment. The microwave scattering temperature sensor has high working frequency, small structure size, and high quality factor (Q), which has little influence on its interference in the background metal environment, and can realize data reading and energy transmission in long distances under harsh environments [14,15,16].

The substrate integrated waveguide (SIW) is a new kind of waveguide structure that can be integrated into a dielectric substrate. Its propagation characteristics are similar to those of rectangular metal waveguides, so the microwave millimeter-wave components and subsystems composed of them have the advantages of high Q value, low radiation loss, strong anti-interference, etc. [9]. As a new type of structural ceramic material, Si_3_N_4_ ceramics has the advantages of higher strength, better high temperature resistance, small thermal expansion coefficient, not easy to generate thermal stress, high temperature creep resistance, and other advantages, showing great application potential in high temperature structural materials such as engines [17].

In this paper, a new type of wireless passive high temperature sensor based on CSRR-SICW and high temperature resistant Si_3_N_4_ ceramics is designed. The microwave scattering technology is used to realize the remote signal monitoring, which solves the problem of the traditional wired sensor transmission line being damaged under high temperatures. A new type of high temperature resistant structural ceramic (Si_3_N_4_ ceramics) was used as the substrate material of the sensor to realize temperature measurement in the range of 25–1000 °C. The complementary split ring resonator and the substrate integrated circular waveguide (CSRR-SICW) structure were used to improve the sensitivity of the sensor and expand the application of the traditional sensor in a polymetallic environment.

## 2. Working Principle and Structure Analysis

**Figure 1:**
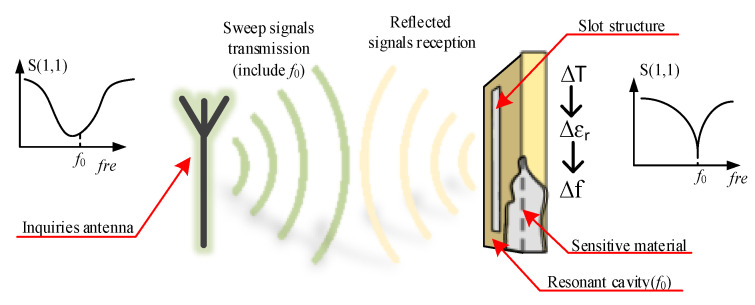
Operating principle of the temperature sensing system.

The measurement principle of wireless passive high temperature parameters based on microwave scattering technology is shown in Figure 1. The system is composed of two parts: an inquiry antenna and a high temperature sensor. The inquiry antenna sends out a sweep signal including the resonant frequency *f*_0_ of the resonant cavity to the temperature sensor which is integrated with the slot antenna and the resonant cavity, the sensor through the slot antenna structure to the incoming signal coupling into the cavity. Among them, only the signal of frequency component *f*_0_ can oscillate inside the sensor and be attenuated gradually, while the other frequency signals are reflected back to the inquiry antenna. When the ambient temperature changes, the dielectric constant of the resonant cavity material changes accordingly, which affects the resonant frequency of the resonant cavity. The resonant frequency of the sensor under different ambient temperatures can be obtained by measuring the return loss of the reflected signal of the sensor received by the inquiry antenna, namely the S(1,1) parameter, and the temperature of the measured environment can be calculated according to the variation of the resonant frequency of the sensor.

**Figure 2:**
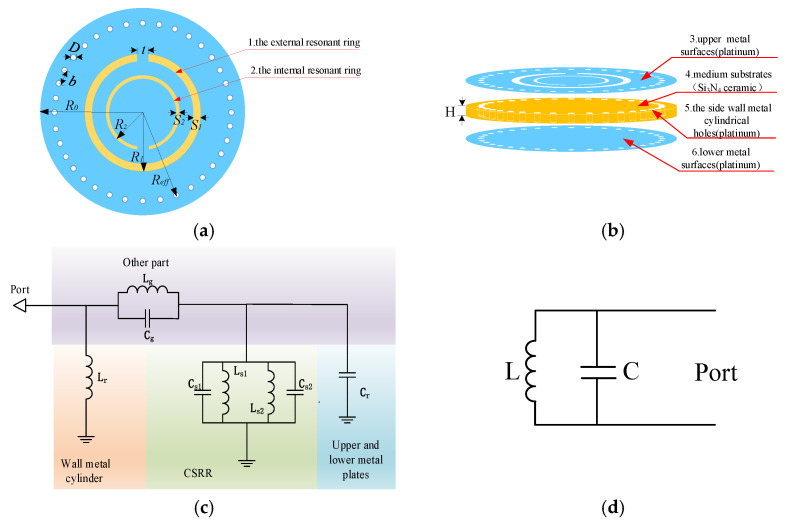
(**a**) Sketch diagram of the front sensor (**b**) Sketch diagram of the side sensor (**c**) Equivalent circuit diagram of the sensor (**d**) Simplified equivalent circuit diagram of the sensor.

As shown in Figure 2a,b, the temperature sensor consists of an SICW resonator and a CSRR structure. The SICW resonator consists of four parts: medium substrate, upper and lower metal surface, and side wall metal cylinder. The medium material of the sensor is high temperature resistant Si_3_N_4_ ceramics. The upper and lower surfaces of the dielectric substrate are covered with a metal platinum paste, and the metal cylindrical through holes in the side walls are connected with the upper and lower metal surfaces. By achieving a metallization aperture, a dielectric substrate can realize the structure of the waveguide, resulting in an electromagnetic field distribution that is nearly the same as that of a conventional waveguide. The upper metal surface etched the CSRR structure. The main function of the CSRR structure is that it can generate a centralized electromagnetic field to improve sensor sensitivity and realize wireless signal transmission.

Where *D* is the diameter of the metal cylindrical hole on the side wall, *b* is the distance between the two adjacent cylindrical hole centers, *R_0_* is the radius of the sensor, *R_eff_* is the distance between the metal cylindrical hole on the side wall and the sensor center, and *H* is the thickness of the sensor, namely the distance between the upper and lower metal surfaces. *R*_1_ is the external radius of the external resonant ring of the CSRR structure, *s*_1_ is the gap width of the external resonant ring, *R*_2_ is the external radius of the internal resonant ring of the CSRR structure, *s*_2_ is the gap width of the internal resonant ring, and *t* is the opening width of the resonant ring of the CSRR structure.

The resonant frequency of the SCIW structure is [11]:

(1) $$f_{0}=\frac{c}{\sqrt{\mu \varepsilon }}\frac{P_{11}}{2\pi R_{eff}}$$

(2) $$R_{eff}=R_{0}-1.08\frac{D^{2}}{b}+0.1\frac{D^{2}}{R_{0}}$$

where *f*_0_ is resonant frequency, *c* is the speed of light, *P*_11_ is the first zero of a first-order Bessel function(*P*_11_ = 2.4048), ε for the dielectric constant of dielectric material, μ for the magnetic permeability of medium material. When the size of the sidewall metal cylinder is *D* < 0.1*λ_g_*, *b* < 4*D* and *D* < 0.2*R_eff_*, the sidewall metal cylinder can be regarded as an ideal electromagnetic wall, and the electromagnetic wave leakage from it can be ignored. To a certain extent, the electromagnetic interference of the external metal environment to the sensor signal can be reduced. When the size of the sensor is fixed, the resonant frequency is determined by the dielectric constant of the dielectric material. The dielectric constant of the sensor material will increase with the temperature increasing accordingly, leading to the decrease of the resonant frequency of the sensor, so as to realize the temperature measurement.

The equivalent circuit of the designed sensor is analyzed, as shown in Figure 2c. The metal cylinder on the side wall of the substrate integrated waveguide structure can be equivalent to a parallel inductor (*L_r_*), and the upper and lower metal plates can be equivalent to a capacitor (*C_r_*). CSRR structure can be equivalent to the parallel connection of two inductors (*L_s_*) and their inter-ring coupling capacitors (*C_s_*), wherein *L_s_*_1_ and *L_s_*_2_ are equivalent circuits of the inner and outer resonant rings, respectively, and the metal walls on both sides of the inner and outer resonant rings are equivalent to *C_s_*_1_ and *C_s_*_2_ in turn. Among them, the equivalent inductance of CSRR structure and the equivalent capacitance of the upper and lower metal surfaces of SICW structure play a dominant role, so other parts of the equivalent circuit can ignore its influence. Then the resonant frequency of the sensor is:

(3) $$f_{0}=\frac{1}{2\pi \sqrt{\frac{L_{s1}+L_{s2}}{L_{s1}L_{s2}}\left(C_{s1}+C_{s2}+C_{r}\right)}}$$

(4) $$C_{r}=\frac{\varepsilon S}{4\pi kd}$$

where ε is the dielectric constant of the medium between the plates, *S* is the opposite area of the capacitor plate, *d* is the distance between the plates, k is the static force constant (k = 8.987551 × 10^9^ N·m^2^/C^2^).

When CSRR structure is determined, *L_s_* and *C_s_* are determined. *C_r_* is determined by the medium material between the upper and lower metal sheets. The dielectric constant of Si_3_N_4_ ceramics increases with the increase of temperature. According to Equation (4), the equivalent capacitance *C_r_* increases, and the resonant frequency of the sensor decreases accordingly. The simplified equivalent circuit of the sensor is shown in Figure 2d. The sensor can be simplified and equivalent to the parallel connection of inductor and capacitor, and the resonant frequency can be simplified to Equation (5).

(5) $$f_{0}=\frac{1}{2\pi \sqrt{LC}}$$

## 3. Simulation and Optimization

In order to improve the transmission efficiency of the sensor and reduce the loss, HFSS software was used to model and simulate the sensor and the inquiry antenna, respectively. The performance of the sensor was judged by the return loss in the response curve, and the optimal size parameters were obtained.

**Figure 3:**
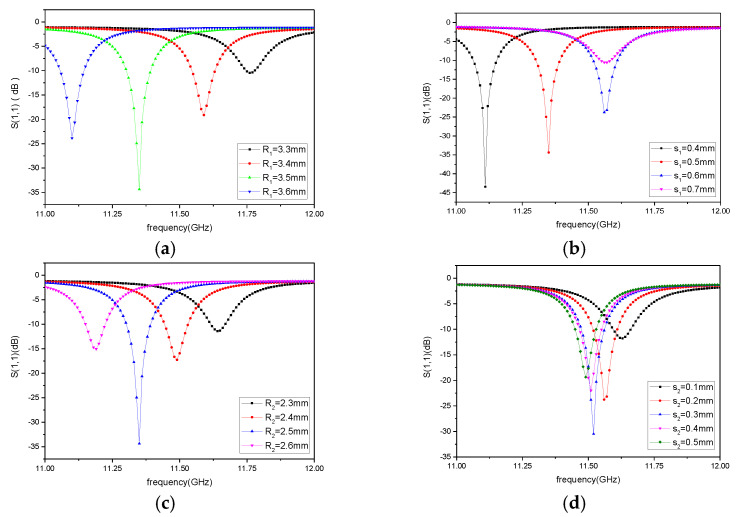
Simulation of complementary split ring resonator (CSRR) Parameter Optimization: (**a**) Radius of CSRR external resonant ring *R*_1_, (**b**) Gap width of CSRR external resonant ring *s*_1_, (**c**) Radius of CSRR internal resonant ring *R*_2_, (**d**) Gap width of CSRR internal resonant ring *s*_2_.

The resonant frequency of the sensor set in this paper is *f*_0_ = 11.5 GHz. The high-temperature resistant ceramic (Si_3_N_4_ ceramics) is used as the sensitive material of the sensor. At room temperature, the dielectric constant is 3.6 and the relative permeability is 0.98. The standard rectangular waveguide is used as the excitation source of the CSRR-SICW sensor. The size of the rectangular waveguide is 20.78 mm × 9.24 mm × 46 mm. Under the condition of satisfying the leak-proof size of the metal cylinder on the side wall, combined with formulas (1) and (2), the dimensional parameters of the sensor are preliminarily calculated as: *R_eff_* = 5.5 mm, *R*_0_ = 7 mm, *D* = 0.5 mm, *H*= 1.1 mm. The number of metal cylinders on the side wall was 36. In order to improve the performance of the substrate integrated waveguide sensor, the external resonant ring radius *R*_1_, the external resonant ring gap width *s*_1_, the internal resonant ring radius *R*_2_, and the internal resonant ring gap width *s*_2_ of the CSRR structure were simulated and analyzed, respectively. The simulation results of parameter optimization are shown in Figure 3.

According to the simulation results, the radius of the CSRR external resonant ring *R*_1_, the width of the gap of the external resonant ring *s*_1_ and the radius of the internal resonant ring *R*_2_ all affect the resonant frequency of the sensor. When *R*_1_ increases, the resonant frequency decreases accordingly. This is because the equivalent capacitance increases with the increase of *R*_1_, leading to the decrease of the resonant frequency of the CSRR structure. When *R*_1_ remains unchanged, when the metal walls on both sides of the external resonant ring become larger, the distance between the two plates of the equivalent capacitor becomes larger, and the equivalent capacitance decreases accordingly. The resonant frequency of the improved CSRR structure increases. According to the simulation results, when the distance between the plates reaches a certain distance, the influence on the equivalent capacitance gradually decreases. Similarly, the influence of the inner circle radius and the gap of the internal resonant ring on the resonant frequency of CSRR structure can be obtained. The resonant frequency of the CSRR structure of the sensor is mainly determined by the radius and the gap of the external resonant ring and the radius of the internal resonant ring; however, the gap of the internal resonant ring mainly plays a role in strengthening. Therefore, the precise regulation of the resonant frequency can be realized by flexibly adjusting the size parameters of the CSRR structure. The structural size of the sensor is shown in Table 1. In order to obtain the quality factor of the sensor, HFSS software was used to model and simulate the designed sensor in the eigen-mode. According to the simulation results, the quality factor of the designed sensor was 1215.93.

**Figure 4:**
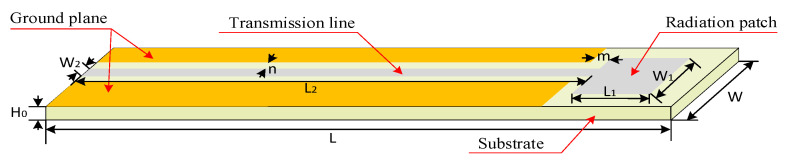
Structure diagram of the inquiry antenna.

The schematic diagram of the designed coplanar waveguide (CPW) antenna is shown in Figure 4. Respectively, *W* and *L* are the width and length of the inquiry antenna, *W***_1_** and *L***_1_** are the width and length of the radiation patch, *W***_2_** and *L***_2_** are microstrip transmission line width and length, *m* and *n* are the spacing widths between the receiving floor, the radiation patch, and the transmission line, respectively. The dimensions of the coplanar waveguide antenna are shown in Table 2.

**Figure 5:**
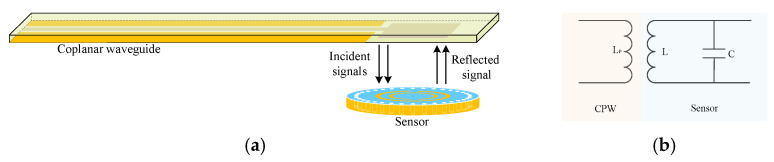
(**a**) Wireless passive sensor model, (**b**) simplified equivalent circuit diagram.

**Figure 6:**
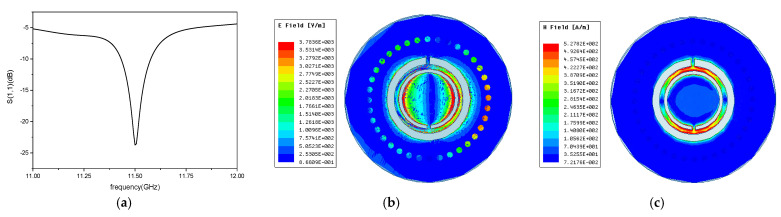
(**a**) Resonant frequency of the sensor, (**b**) electric field of the sensor, (**c**) magnetic field distribution of the sensor.

The previously simulated CSRR-SICW high temperature sensor is placed under the CPW antenna to receive and send signals. The model and simplified equivalent circuit diagram is shown in Figure 5. The resonant frequency of the sensor at room temperature is 11.5 GHz. The distribution of electric field and magnetic field of the sensor is shown in Figure 6, indicating that the strongest electromagnetic field is mainly distributed around the CSRR structure.

## 4. High Temperature Experimental Test

### 4.1. Preparation of Sensor

**Figure 7:**
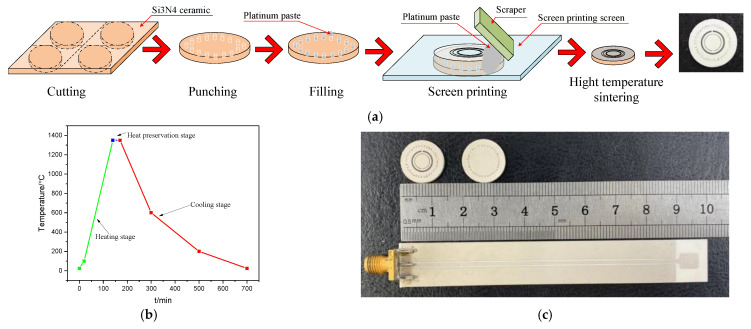
(**a**) Sensor manufacturing process, (**b**) sensor sintering curve, (**c**) high temperature sensor and inquiry antenna.

The substrate material of the sensor is Si_3_N_4_ ceramics. First, the purchased ceramic pieces are cut into discs with a diameter of 14 mm. By using laser drilling technology, the cut circular substrate is placed under the laser drilling machine for drilling, and the diameter of the through hole is 0.5 mm, and the sidewall cylindrical through-hole array of SICW structure is realized. Because the platinum pulp can withstand a high temperature environment of 1800 °C and the material properties are relatively stable in the high temperature environment, we use the platinum pulp as the surface metal and through hole filling material of the sensor. We then put the prepared substrate into the micro-hole filling machine and injected the platinum pulp, so that the upper and lower surfaces can be connected, and placed in an environment of 100 °C drying for 30 min. After that, the dust on the upper and lower surfaces of the substrate was wiped with 99% anhydrous alcohol and dust-free paper, and the surfaces of the SICW and the slot antenna were brushed by screen printing technology, respectively, with printing thickness of 20 μm. Finally, the sensor was placed in a muffle furnace for sintering; the sintering curve is shown in Figure 7b. High temperature sintering can remove organic solvents from the paste, so that a dense platinum film can be formed on the Si_3_N_4_ ceramics substrate. The final high temperature sensor and inquiry antenna are shown in Figure 7c.

### 4.2. High Temperature Test of the Sensor

**Figure 8:**
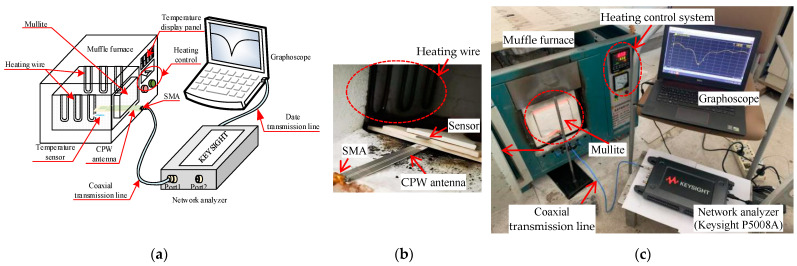
(**a**) High temperature environment test platform diagram, (**b**) experimental test diagram, (**c**) experimental test device.

In order to verify the temperature sensing performance of the sensor, the high temperature test platform built is shown in Figure 8. Temperature testing mainly includes computers, network analyzer (Keysight P5008A, Keysight Technologies, Santa Rosa, CA, USA), coaxial transmission lines, CPW antennas, sensors, and a high temperature muffle furnace.

In the test process, the sensor and inquiry antenna were placed in the muffle furnace, and the mullite was used to prevent the cold end of the inquiry antenna from being damaged by the high temperature environment in the furnace. The cold end of the inquiry antenna is connected with the network analyzer through the coaxial transmission line, which is used to transmit electromagnetic signals to the sensor and receive reflected signals from the sensor. The network analyzer is connected to the display to display and save the test data.

**Figure 9:**
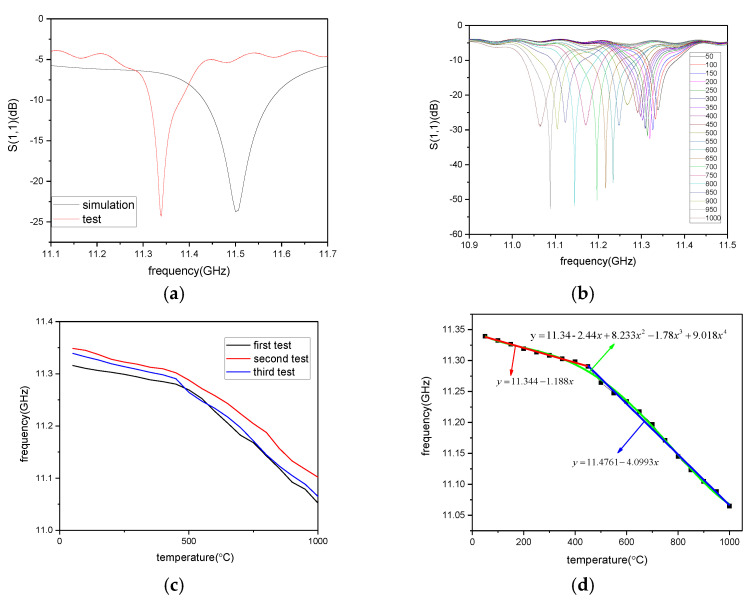
(**a**) Comparison of simulation and test at room temperature, (**b**) Resonant frequency curve of the sensor at different temperatures tested, (**c**) Repeat of temperature test results, (**d**) Quaternary polynomial fitting curve and Piecewise linear fitting curve of high temperature sensor resonant frequency variation curve with temperature.

At room temperature, the test results of the sensor are slightly different from the simulation resonant frequency, as shown in Figure 9a. This result may be caused by errors in the sensor machining process, or the sensor is in an ideal environment during simulation. The temperature test in this paper starts from 50 °C, and then the temperature rise test is carried out step by step, and the data is recorded every 50 °C. To ensure repeatability of the sensor, the maximum temperature was raised to 1000 °C. The measured resonant frequency of the high temperature sensor decreases with the increase of temperature, and the curve of change is shown in Figure 9b. In the figure, S(1,1) is the self-reflection coefficient of the inquiry antenna, frequency is the trough point represents the resonant frequency of the sensor. The resonant frequency of the sensor is 11.3392 GHz at 50 °C, 11.2904 GHz at 450 °C, and 11.0648 GHz at 1000 °C. By extracting the resonant frequency of the trough point from the curve directly tested, the change curve of the resonant frequency of the high temperature sensor at 50–1000 °C is finally obtained. The resonant frequency decreases with the increase in temperature, which is consistent with the theory.

The temperature test was repeated three times, and the test results were shown in Figure 9c. According to the results, the designed sensor could better reproduce the experimental results. After preliminary analysis and processing of the data, the quaternion polynomial fitting curve is shown as a green line in Figure 9d.The fitting curve is expressed in Equation (6). However, the quaternion polynomial is only suitable for small range of calculation, not speculative. After further processing of the test data, the test results show that the resonant frequency has different change rates with the increase of temperature in the temperature range of 50–450 °C and 450–1000 °C. Linear fitting was conducted for the data; the fitting curves were shown as the red line and blue line in Figure 9d, respectively, and their expressions are shown in Equation (7).

(6) $$y=11.34-2.44x+8.233x^{2}-1.78x^{3}+9.018x^{4}$$

(7) $$y=\{\begin{array}{c} 11.344-1.188x,0<x\leq 450\\ 11.4761-4.0993x,450<x\leq 1000 \end{array}$$

According to analysis of the test data, the resonant frequency of the sensor changes linearly at different temperatures. The sensitivity of the resonant frequency of the sensor is 123 kHz/°C at 50–450 °C, and 417 kHz/°C at 450–1000 °C. The average test sensitivity of the sensor is 289 kHz/°C. According to the literature [17], ion displacement polarization is the main factor affecting the variation of dielectric constant with temperature. The polarization of the Si_3_N_4_ dipole moment decreases with the increase of temperature, which results in the increase of the dielectric constant of Si_3_N_4_ ceramics. According to Equation (2), the resonant frequency is affected not only by the dielectric constant of the dielectric material, but also by the metal cylinder on the side wall. The thermal expansion coefficient of the Si_3_N_4_ ceramics increases with the increase of temperature, which results in the increase of the relative position of the metal column on the side wall. This explains why the sensor is more sensitive at 450–1000 °C.

Table 3 shows a comparison between the sensor designed in this paper and the sensor previously reported. The sensor designed in this paper has the following advantages:

1. The temperature range is large enough to test relatively high temperatures;
2. The sensor has high sensitivity. In the temperature test, it was found to have a large frequency change, and the resonant frequency signal is stronger and easier to be captured;
3. The smaller size of the sensor makes it easier to mount inside the engine, such as on the metal blade surface.

## 5. Conclusions

In this paper, a wireless passive high temperature sensor based on Si_3_N_4_ ceramics is proposed. The sensor consists of an improved CSRR and SICW structure, and its equivalent circuit is analyzed. The dimensional parameters of the sensor were optimized and determined by theoretical calculation and HFSS software simulation. The high temperature test results show that the resonant frequency of the high temperature sensor decreases from 11.3392 GHz to 11.0648 GHz in the range of 50–1000 °C. When the temperature is 50–450 °C and 450–1000 °C, the sensitivity of the sensor is 123 kHz/°C and 417 kHz/°C, respectively, and the average test sensitivity is 289 kHz/°C. Through analysis, the sensor designed in this paper was found to be small in size, easy to be installed on metal blade surfaces, and had higher sensitivity at 450 °C or above. The experimental results verify the rationality of the design and feasibility of the CSRR-SICW wireless passive high temperature sensor based on Si_3_N_4_ ceramics, and show its application potential in the harsh environment of ultra-high temperatures.

## Figures and Tables

**Table 1:** Dimensional parameters of the optimized sensor (unit: mm).

*R_eff_*	*R*_0_	*D*	*b*	*R*_1_	*s*_1_	*R*_2_	*s*_2_	*H*
5	7	0.5	2	3.5	0.6	2.5	0.2	1.1

**Table 2:** Structure dimensions of the CPW antenna (unit: mm).

*W*	*L*	*W*_1_	*L*_1_	*W*_2_	*L*_2_	*n*	*m*	*H*_0_
14	100	4.7	6.1	1	90	0.5	1.5	1

**Table 3:** Parameters comparison of different types of temperature sensors.

Sensor Type	Sensor Size	Operating Frequency	Measuring Range	Sensitivity
CSRR-SICW sensor	*R* = 7 mm, *h* = 1 mm	Around 11.34 GHz	25–1000 °C	289 kHz/°C
SIW sensor [10]	35 mm × 35 mm × 1 mm	Around 2.27 GHz	25–1200 °C	197 kHz/°C
Micro-strip patch sensor [18]	44.58 mm × 68 mm × 1 mm	Around 2.2 GHz	25–700 °C	104.7 kHz/°C
LC sensor [19]	150 mm × 21 mm	Around 55 MHz	25–1200 °C	0.009 dB/°C
SAW sensor [11]	-	Around 226.3 MHz	25–600 °C	-
